# Context-specific applications of CARM1 inhibitors: functional profiles of EZM2302 and TP-064

**DOI:** 10.1186/s10020-025-01388-y

**Published:** 2025-10-31

**Authors:** Yena Cho, Yong Kee Kim

**Affiliations:** 1 Muscle Physiome Research Center and Research Institute of Pharmaceutical Sciences, Sookmyung Women’s University, Seoul, 04310 Republic of Korea; 2 College of Pharmacy, Sookmyung Women’s University, Seoul, 04310 Republic of Korea

**Keywords:** CARM1, Arginine methylation, TP-064, EZM2302, Autophagy

## Abstract

**Background:**

Coactivator-associated arginine methyltransferase 1 (CARM1) regulates diverse cellular processes—including transcription, cell cycle progression, metabolism, and autophagy—through asymmetric dimethylation of both histone and non-histone substrates. Although TP-064 and EZM2302 both inhibit CARM1, they may elicit distinct biological effects.

**Methods:**

We employed immunoblotting, subcellular fractionation, histone extraction, chromatin immunoprecipitation assay, quantitative PCR, and confocal microscopy to compare the effects of TP-064 and EZM2302. Substrate methylation and autophagic responses were evaluated under nutrient-deprived conditions.

**Results:**

Both TP-064 and EZM2302 inhibited CARM1-dependent methylation of non-histone substrates, including p300, GAPDH, and DRP1. However, TP-064 markedly reduced nuclear histone methylation marks H3R17me2a and H3R26me2a, whereas EZM2302 had minimal effect on these epigenetic modifications. Reflecting this differential impact, TP-064—but not EZM2302—suppressed transcription of autophagy-related genes and impaired LC3 lipidation and puncta formation under glucose deprivation. Consequently, TP-064 sensitized cells to energy stress by disrupting autophagic flux. These findings indicate that TP-064 inhibits both nuclear and cytoplasmic functions of CARM1, while EZM2302 selectively targets non-histone methylation events.

**Conclusion:**

Our study reveals fundamental mechanistic differences between TP-064 and EZM2302 in regulating CARM1 substrates and downstream pathways. This substrate-selective inhibition has important implications for experimental design and therapeutic development, underscoring the need for context-specific selection of CARM1 inhibitors in both basic research and precision medicine.

**Supplementary Information:**

The online version contains supplementary material available at 10.1186/s10020-025-01388-y.

## Introduction

Coactivator-associated arginine methyltransferase 1 (CARM1), first identified in 1999 (Chen et al. 1999), has since emerged as a multifunctional regulator involved in transcription, cell cycle progression, DNA damage response, oncogenesis, autophagy, and metabolism (Hwang et al. 2021; Santos et al. 2023; Cho et al. 2025a; Cho et el. 2025b). CARM1 belongs to the family of protein arginine methyltransferases (PRMTs), which catalyze the post-translational methylation of arginine residues on histone and non-histone proteins using S-adenosylmethionine (SAM) as a methyl donor. PRMTs are classified into three major types based on the methylarginine products they generate: Type I enzymes produce monomethylarginine (MMA) and asymmetric dimethylarginine (ADMA), Type II enzymes generate MMA and symmetric dimethylarginine (SDMA), and Type III enzymes exclusively forms MMA (Hwang et al. 2021). As a Type I PRMT, CARM1, also known as PRMT4, specifically catalyzes the formation of MMA and ADMA, thereby regulating protein function through methylation-dependent modulation of protein stability, subcellular localization, and protein–protein interactions.

These methylation events underscore the broad biological significance of CARM1 and position it as a key epigenetic and signaling regulator. Advances in genetic and chemical biology have enabled detailed dissection of CARM1’s physiological and pathological roles. Genetic models, including CARM1 knockout (KO) and knock-in mice, have elucidated its essential roles in development and tissue homeostasis (Yadav et al. 2003; Kim et al. 2010; Santos et al. 2023). More recently, the development of highly selective small-molecule inhibitors has provided powerful tools for the temporal and reversible regulation of CARM1 activity in both cellular and animal systems. Among these, TP-064 and EZM2302 have gained particular attention for their potent and selective inhibition of CARM1 (Fig. S1) (Drew et al. 2017; Nakayama et al. 2018). Despite their similar inhibitory potencies, TP-064 and EZM2302 may exhibit distinct pharmacological profiles due to differences in their binding mechanisms. TP-064 binds cooperatively with SAM to the catalytic domain of CARM1, inducing conformational changes that alter substrate recognition and inhibit its enzymatic activity in a non-competitive manner (Nakayama et al. 2018). In contrast, EZM2302 stabilizes an inactive CARM1–S-adenosylhomocysteine (SAH) complex, thereby preventing substrate access and inhibiting methyltransferase activity (Drew et al. 2017). Although EZM2302 has been reported to fail in reducing histone arginine methylation in cell-based assays (Drew et al. 2017), we delineate here a key functional distinction between TP-064 and EZM2302 in regulating histone methylation and transcriptional programs, ultimately leading to divergent biological outcomes, particularly in the context of autophagy and apoptosis.

## Materials and methods

### Chemicals, plasmids, and antibodies

EZM2302 (HY-111109) and TP-064 (HY-114965) were obtained from MedChemExpress (Monmouth Junction, NJ, USA). GFP-LC3 (#21073) plasmid was purchased from Addgene (Watertown, MA, USA). The following antibodies were used for immunoblotting or immunoprecipitation: Actin (Santa Cruz Biotechnology, Dallas, TX, USA, sc-47778), AMPK (Cell Signaling Technology, Danvers, MA, USA, #5832), p-AMPK (Cell Signaling Technology, #2535) CARM1 (Bethyl Laboratories, Montgomery, TX, USA, A300-421 A), DRP1 (BD Biosciences, Franklin Lakes, NJ, USA, #611113), GAPDH (Santa Cruz Biotechnology, sc-25778), histone H3 (Cell Signaling Technology, #9715), H3R2me2a (Sigma-Aldrich, St. Louis, MO, USA, 07–585), H3R17me2a (Abcam, Cambridge, UK, ab8284), H3R26me2a (EpigenTek, Farmingdale, NY, USA, #A-3707), LC3 (Cell Signaling Technology, #12741), p300 (abcam, ab14984), PARP (Santa Cruz Biotechnology, sc-8007), and HRP-conjugated secondary antibodies (Jackson ImmunoResearch Laboratories, West Grove, PA, USA, #111-035-003, #115-035-003). The ADMA^5825^ and NFIBme2a antibodies, widely used for detecting CARM1 substrates (Gao et al. 2023; Cho et al. 2024a; Cho et al. 2025a), were kindly provided by Dr. Mark T. Bedford (University of Texas MD Anderson Cancer Center).

### Cell culture and transfection

Mouse embryonic fibroblast (MEF; CARM1 wild type and KO) cells were generously provided by Dr. Mark T. Bedford (University of Texas MD Anderson Cancer Center). MDA-MB-468, BT-20, HCC1143, HCC1806, HCC1937, HCC1395, MDA-MB-231, and HEK293T cells were obtained from American Type Culture Collection (Manassas, VA, USA). All cells were cultured in RPMI-1640 (HyClone, Logan, UT, USA) or DMEM (high glucose; HyClone) supplemented with 10% fetal bovine serum (HyClone) and 100 units/mL penicillin/streptomycin (HyClone). For glucose starvation experiments, DMEM (no glucose; Gibco, Grand Island, NY, USA), with the same supplements, was used. All cells were maintained at 37 °C in a humidified incubator with 5% CO_2_. Transfections were performed using TransIT-X2 (Mirus Bio, Madison, Wisconsin, USA), following the manufacturer’s instructions.

### Immunoblotting and Immunoprecipitation

Cells were lysed using RIPA lysis buffer (50 mM Tris-HCl [pH 8], 150 mM NaCl, 0.5% sodium deoxycholate, 0.1% sodium dodecyl sulfate, and 1% Triton X-100) supplemented with a 1× protease and phosphatase inhibitor cocktail (Roche, Basel, Switzerland). Sonication was used to lyse the cells, followed by centrifugation at 16,000 ×*g* for 10 min at 4 °C. After lysate centrifugation and protein quantification, immunoprecipitation was performed using 1 µg of the appropriate antibody per 1 mg of total protein, followed by overnight incubation at 4 °C with rotation. Antibody-protein complexes were captured using protein A/G Sepharose beads (Santa Cruz Biotechnology) for 2 h at 4 °C on a rotator. Following two washes with lysis buffer, the complexes were eluted and separated using SDS-PAGE. The separated proteins were transferred onto a PVDF membrane (Millipore, Billerica, MA, USA) and blocked with 5% skim milk in 0.1% Tween 20/Tris-buffered saline (TBS-T) for 1 h at room temperature. The membrane was then incubated with a primary antibody overnight at 4 °C. After washing three times with TBS-T, the membrane was incubated with HRP-conjugated secondary antibody for 1 h at room temperature. The signal was detected using an ECL western blotting substrate (Advansta, Menlo Park, CA, USA).

### Subcellular fractionation

Cells were scraped from the culture dish, washed with PBS, and collected by centrifugation. The cell pellets were gently resuspended in hypotonic lysis buffer (10 mM HEPES [pH 7.5], 10 mM MgCl_2_, and 20 mM KCl) and incubated on ice for 10 min. Following incubation, 0.5% NP-40 was added, and the homogenates were centrifuged at 700 ×*g* for 10 min at 4 °C. The supernatant containing the cytoplasmic fraction was transferred to a new tube. The pellet containing the nuclear fraction was resuspended in RIPA lysis buffer, sonicated, and centrifuged at 16,000 ×g for 10 min at 4 °C. The resulting supernatant was transferred to a new tube.

### Histone extraction

Cells were lysed with RSB buffer (10 mM Tris-HCl [pH 7.6], 10 mM NaCl, and 3 mM MgCl_2_) and centrifuged at 600 × g for 5 min. The supernatant was discarded, and the pellet was resuspended in RSB buffer containing 0.5% NP-40. After incubation, the homogenates were centrifuged at 600 × g for 5 min. The pellet containing nuclei was resuspended in 5 mM MgCl_2_, and an equal volume of 0.8 M HCl was added. Histones were extracted on ice for 20 min and centrifuged at 17,000 × g for 10 min. The supernatant was transferred to a new tube, and histones were precipitated with 50% trichloroacetic acid. The mixture was centrifuged at 12,000 × g for 20 min. The pellet was washed once with acetone-0.3 M HCl and twice with acetone, then dried and resuspended in water, followed by the addition of 1.5 M Tris-HCl [pH 8.8].

### In vitro methylation assay

As previously described (Cho et al., 2024a), 0.5 µg recombinant histone octamer (Active Motif, Carlsbad, CA, USA) was mixed with 1 µM SAM in a tube, followed by the addition of 0.1 µg GST-CARM1 in the presence or absence of 10 nM CARM1 inhibitors. After incubation for 1 h at 37 °C, the methylation reaction was terminated by adding protein sample loading buffer and heating the mixture at 95 °C for 5 min. In certain experiments, nuclear and cytoplasmic fractions isolated from CARM1 KO MEF cells were used to analyze CARM1 substrate proteins. These fractions were prepared using the subcellular fractionation method described above.

### Real-time quantitative reverse transcription PCR (qRT–PCR)

Total RNA was extracted using TRIzol reagent (Bioline, London, UK). Chloroform was added to the cell lysate, which was vortexed and incubated on ice for 10 min. The mixture was centrifuged at 16,000 ×*g* for 15 min at 4 °C, and the colorless aqueous phase containing RNA was transferred to a new tube. Isopropanol was added to an equal volume, and the samples were incubated for 10 min at room temperature. After centrifugation at 16,000 ×*g* for 10 min, the RNA pellet was washed with 70% ethanol, dried, and resuspended in nuclease-free water. cDNA synthesis was performed using the SensiFAST cDNA Synthesis Kit (Bioline). mRNA expression was analyzed using the QuantStudio 3 Real-Time PCR System (Applied Biosystems, Foster City, CA, USA), the SensiFAST SYBR No-ROX Kit (Bioline), and the ΔΔCT method. The reaction conditions were: cDNA synthesis at 40 °C for 60 min, reverse transcriptase inactivation at 95 °C for 5 min, and PCR cycling at 95 °C for 10 s, 58 °C for 20 s, and 72 °C for 20 s (40 cycles). Relative mRNA expression levels were calculated using the ΔΔCT method, with each target gene normalized to the expression of *Actb*. Primer sequences used were as follows: 5’-ACCTTCTACAATGAGCTGCG-3’ and 5’-CTGGATGGCTACGTACATGG-3’ for *Actb*; 5’-TCCGTGCCATCACATACACA-3’ and 5’-TAAGACTGCTGTGGGGCTGA-3’ for *Atg12*; 5’-CCAGGCTCGACTTGGAGAAAA-3’ and 5’-AGATTTCCACACACATAGATCGC-3’ for *Atg13*; 5’-AGCGGTGATTTCGTCTATTTCG-3’ and 5’-GCTGTTCAATCCTCATCTTGCAT-3’ for *Atg14*; 5’-GACATGTTTTCTGACGGCAAC-3’ and 5’-AAGTCCAATGTCCAGCCC-3’ for *Bax*, 5’-GTGGATGACTGAGTACCTGAAC-3’ and 5’-GCCAGGAGAAATCAAACAGAGG-3’ for *Bcl2*; 5’-CACTGCTCTGTCTTGTGTAGGTTG-3’ and 5’-TCGTTGTGCCTTTATTAGTGCATC-3’ for *Map1lc3b*.

### Chromatin immunoprecipitation (ChIP) assay

The ChIP assay was conducted as previously described (Hwang et al. 2019) using the ChIP Assay Kit (Millipore, 17–371). Chromatin fragmentation was performed with the ultrasonic liquid processor (Sonics & Materials, Newtown, CT, USA) at 20% amplitude for 30 cycles of 3 s, resting for 3 s to obtain an average fragment size between 200 and 500 bp. ChIP and input DNA were then purified and analyzed for qRT–PCR analysis. The following primers were used in ChIP assays. 5’-AGCCAGTGGGATATTGGTCT-3’ and 5’-AGAGCCTGCGGTACCCTAC-3’ for *Map1lc3b*; 5’-GAGACGCCATGATGATCTGA-3’ and 5’-GCCAAGGAGTGTGGGAAGTA-3’ for *Atg14*.

### Cell viability assay

After treatment with 1 µM TP-064 or EZM2302 for 72 h, the cells were further incubated for an additional 12 h in media supplemented with or without glucose. Subsequently, MTT (0.5 mg/mL) was added to each well and incubated for 2 h. The medium was then discarded, and 120 µL of DMSO was added to dissolve the formazan crystals. The absorbance was measured at 590 nm using Epoch Microplate Spectrophotometer (BioTek, Winooski, VT, USA).

### Long-term live cell imaging

Cells were seeded in 12 well plates and allowed to adhere for 24 h. Following pretreatment with 1 µM TP-064 or EZM2302 for 24 h, cells were incubated for an additional 36 h in media with or without glucose. Plates were placed in the IncuCyte ZOOM (Essen BioScience, Ann Arbor, MI, USA) at the Chronic and Metabolic Diseases Research Center, Sookmyung Women’s University. Phase-contrast images were acquired every 3 h at 10× magnification, and cell confluence was quantified using the IncuCyte software’s integrated cell counting algorithm. Data were normalized to baseline and plotted as growth curves.

### Confocal microscopy

Cells plated on coverslips were fixed with 4% paraformaldehyde and permeabilized with 0.5% Triton X-100 for 15 min. After washing with PBS, cells were stained with 4′,6-diamidino-2-phenylindole (DAPI) (Thermo Fisher Scientific, Waltham, MA, USA) for 15 min at 200 nM concentration in the dark. The cells were washed with PBS and mounted onto glass slides. The images were visualized and analyzed using Zeiss LSM 710 Confocal Microscope (Carl Zeiss, Oberkochen, Germany) and images were analyzed using ZEN.

### Statistical analysis

All statistical analyses were performed using the Prism software (GraphPad). Data are representative of independent experiments and have been presented as mean ± standard deviation (*n* ≥ 3). Data from two groups were compared using unpaired *t*-test for independent samples. Statistical significance was set at *p* < 0.05. *p < 0.05, **p < 0.01, and ***p < 0.001.

## Results and discussion

### Differential effects of CARM1 inhibitors on histone methylation

**Fig. 1:**
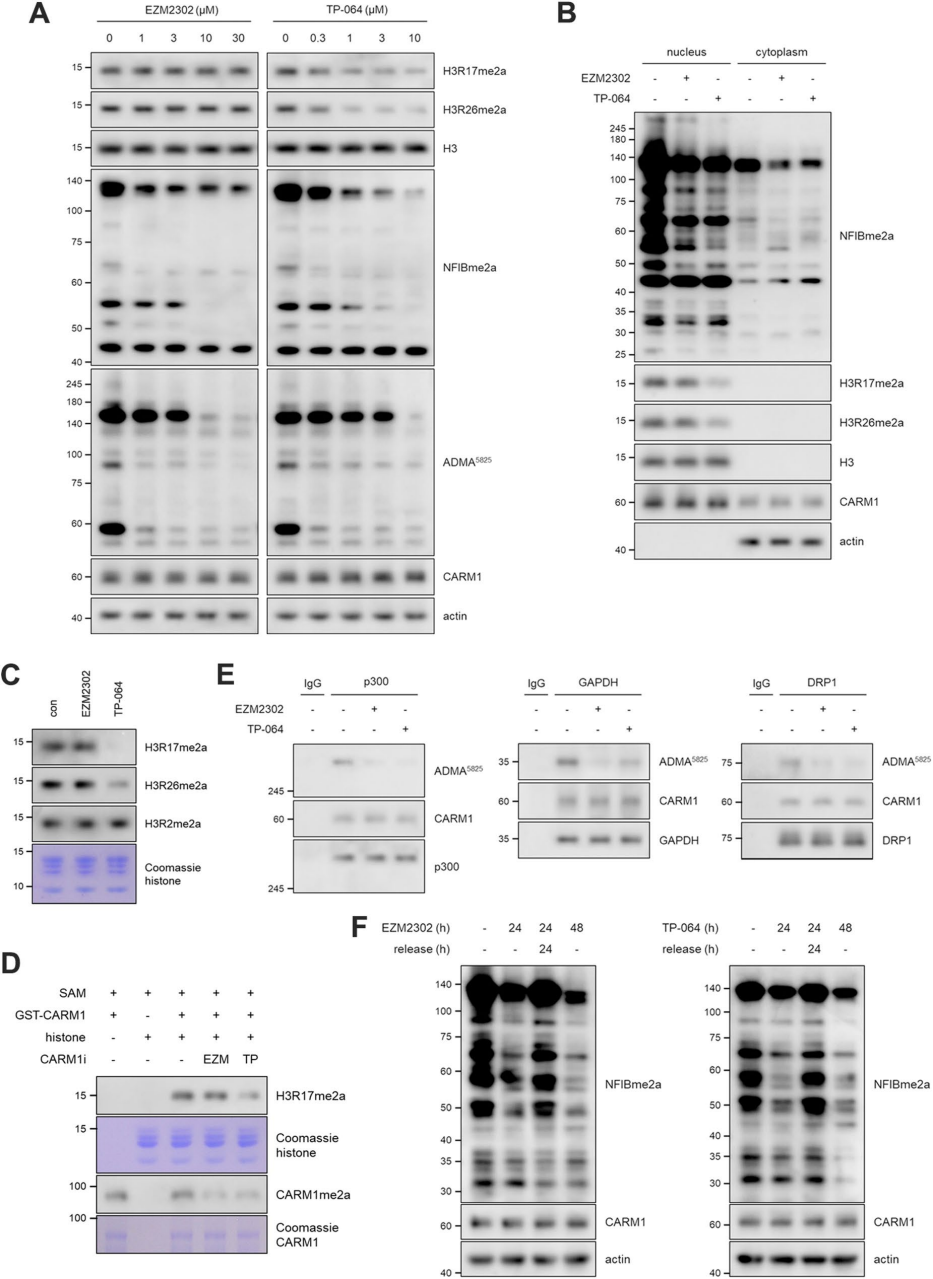
Differential effects of CARM1 inhibitors on histone methylation. (**A**–**C**) Western blots of whole cell lysates (**A**), nuclear and cytoplasmic fractions (**B**), and histone fractions (**C**) in MEF cells treated with 1 µM EZM2302 or TP-064 for 72 h. (**D**) In vitro methylation assay was performed using 1 µM SAM, 10 nM inhibitor (EZM2302 or TP-064), and proteins (histones and GST-CARM1). The reaction mixture was incubated at 37 °C for 1 h. (**E**) IP-western blots of CARM1 substrates. After treatment with CARM1 inhibitors (1 µM, 72 h), the cell lysates were immunoprecipitated using anti-p300, GAPDH, or DRP1 antibody. (**F**) MEF cells were treated with EZM2302 or TP-064 for 24 h, followed by an additional 24 h incubation in fresh media before analyzing CARM1 substrate levels by western blot. All experiments were independently repeated two or three times, and representative blots are shown

CARM1 is well known to catalyze asymmetric dimethylation of histone H3 at arginine 17 (H3R17me2a) and arginine 26 (H3R26me2a), both of which are associated with transcriptional activation (Wu et al. 2012). Transient knockdown of CARM1 markedly reduced H3R17me2a levels (Fig. S2). However, despite effectively inhibiting CARM1 activity—as evidenced by decreased methylation of non-histone substrates detected using substrate-specific antibodies ADMA^5825^ and NFIBme2a—EZM2302 and TP-064 exhibited divergent effects on histone methylation (Fig. 1A). TP-064 potently reduced both H3R17me2a and H3R26me2a levels (Fig. 1A) and this selective inhibition of histone methylation was further confirmed through nuclear fractionation experiments (Fig. 1B). In contrast, EZM2302 failed to alter either histone mark (Fig. 1A and B), indicating its limited effect on nuclear histone substrates. Notably, neither inhibitor affected H3R2me2a levels—a modification primarily catalyzed by PRMT6 (Cheng et al. 2020)—further supporting the specificity of both compounds for CARM1 (Fig. 1C). In vitro methylation assays consistently demonstrated distinct effects of TP-064 and EZM2302 on histone methylation. TP-064 effectively inhibited CARM1-mediated H3R17me2a, whereas EZM2302 showed no inhibitory effect on this histone substrate (Fig. 1D). As a positive control, CARM1 automethylation (Kuhn et al. 2010) was assessed and found to be markedly suppressed by both TP-064 and EZM2302 (Fig. 1D), confirming their ability to target CARM1. Moreover, in vitro assays using lysates from CARM1 KO MEF cells demonstrated that both inhibitors reduced methylation signals in nuclear and cytoplasmic fractions (Fig. S3). However, only TP-064 induced a substantial decrease in H3R17me2a levels (Fig. S3), reinforcing its selective inhibitory activity toward histone substrates. Importantly, both TP-064 and EZM2302 suppressed the methylation of non-histone substrates such as p300 (Lee et al. 2011) and DRP1 (Cho et al. 2024a; Cho et al. 2024b), play critical roles in transcriptional coactivation and mitochondrial fission, respectively (Fig. 1E and S4). However, TP-064 exhibited relatively weaker inhibition of GAPDH methylation (Zhong et al. 2018) compared to EZM2302 (Fig. 1E), suggesting differences in substrate selectivity between the two compounds. Antibody-based detection using ADMA^5825^ and NFIBme2a further confirmed the reduction of these non-histone methylation events across multiple breast cancer cell lines (Fig. S5). While EZM2302 robustly suppressed non-histone substrate methylation, it left histone methylation largely unaffected (Fig. S5). Meanwhile, both compounds were shown to allow restoration of substrate methylation levels to baseline within 9 h following inhibitor withdrawal (Fig. S6), suggesting that their inhibitory effects are reversible at the cellular level (Fig. 1F, S6, and S7). Taken together, these findings suggest that TP-064 exerts broader nuclear effects by targeting both histone and non-histone substrates, whereas EZM2302 preferentially inhibits non-histone methylation events, indicating distinct substrate-selective mechanisms of action.

### Differential regulation of autophagy by CARM1 inhibitors

**Fig. 2:**
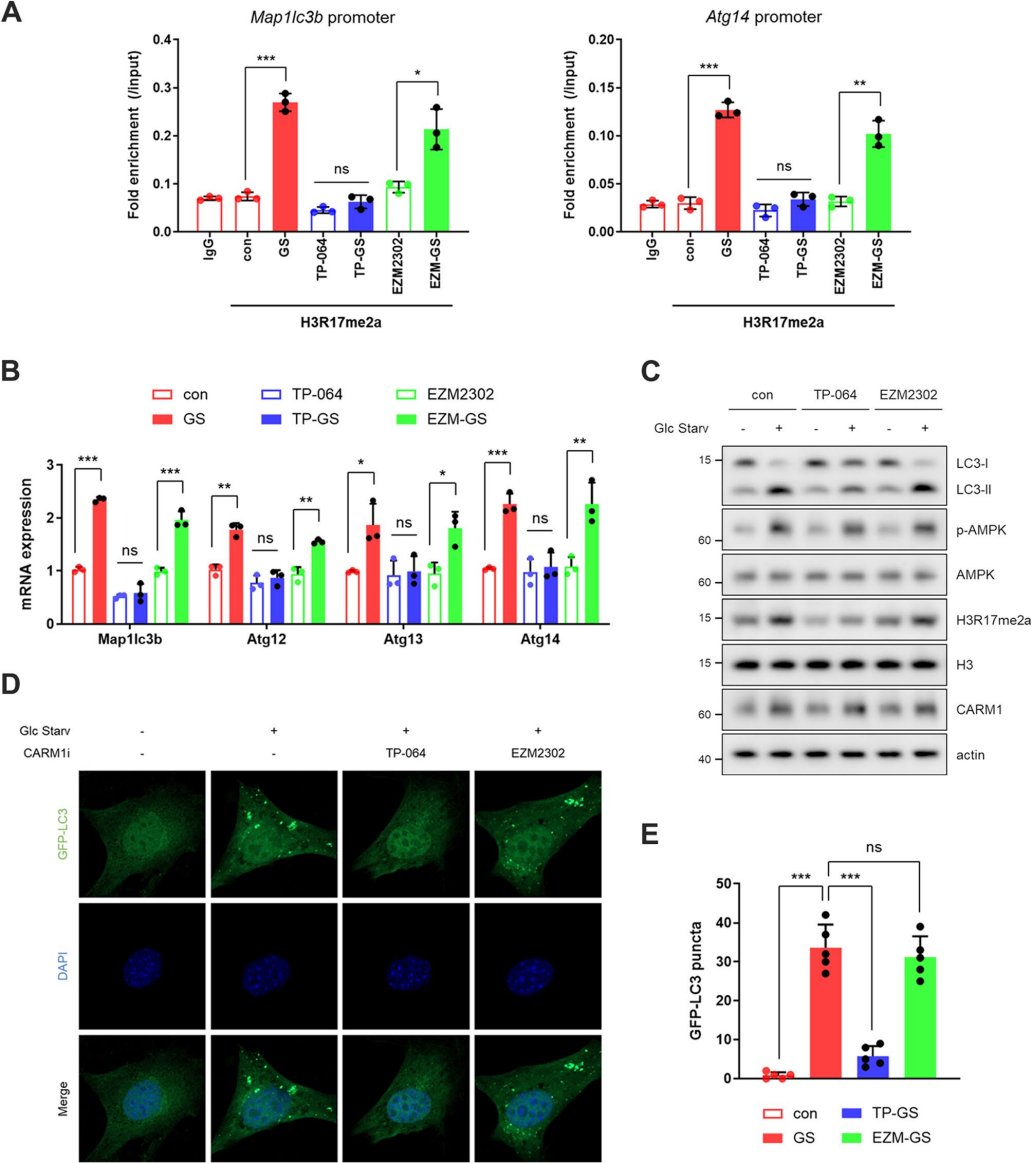
TP-064, but not EZM2302, suppresses energy stress-induced autophagy. (**A**-**E**) ChIP assays of H3R17me2a on *Map1lc3b* and *Atg14* promoters (*n* = 3) (**A**), mRNA expression levels of autophagy-related genes (*n* = 3) (**B**), protein levels of LC3-I and LC3-II (*n* = 3) (**C**), representative confocal images showing GFP-LC3 puncta (**D**), and quantification of GFP-LC3 puncta per cell (*n* = 5) (**E**) in MEF cells treated with CARM1 inhibitors under 12 h glucose deprivation. ‘n’ indicates the number of independent experiments performed

Autophagy represents a critical pathway differentially regulated by these inhibitors. Under nutrient-deprived conditions, CARM1 promotes transcription of autophagy and lysosomal genes *via* H3R17me2a deposition at their promoters (Shin et al. 2016; Yu et al. 2020). ChIP assays revealed that TP-064 significantly suppressed H3R17 methylation at the promoters of autophagy-related genes (*Map1lc3b* and *Atg14*), whereas EZM2302 had no detectable effect (Fig. 2A). Consistent with its impact on histone methylation, TP-064 suppressed the expression of autophagy-related genes (Fig. 2B) and impaired autophagic flux (Fig. 2C and E), as evidenced by decreased LC3 lipidation (LC3-II) and puncta formation under glucose deprivation. In contrast, EZM2302 showed minimal effects on these autophagy markers (Fig. 2A and E), supporting the notion that its mechanism of action is largely independent of histone methylation and nuclear transcriptional remodeling. Additionally, we assessed whether TP-064 or EZM2302 altered AMPK activation, an upstream regulator of starvation-induced autophagy (Egan et al. 2011; Hardie et al. 2012). Neither affected AMPK phosphorylation under glucose deprivation (Fig. 2C), suggesting that the observed differences in autophagy are mediated through AMPK-independent, transcription-dependent mechanisms. Taken together, these findings suggest a model in which TP-064 modulates chromatin dynamics and transcriptional programs *via* SAM-cooperative inhibition of histone methylation, whereas EZM2302 predominantly regulates cytoplasmic functions by targeting non-histone substrates. This divergence has important implications for basic research and therapeutic development.

### TP-064, but not EZM2302, sensitizes to energy stress by inhibiting autophagy

**Fig. 3:**
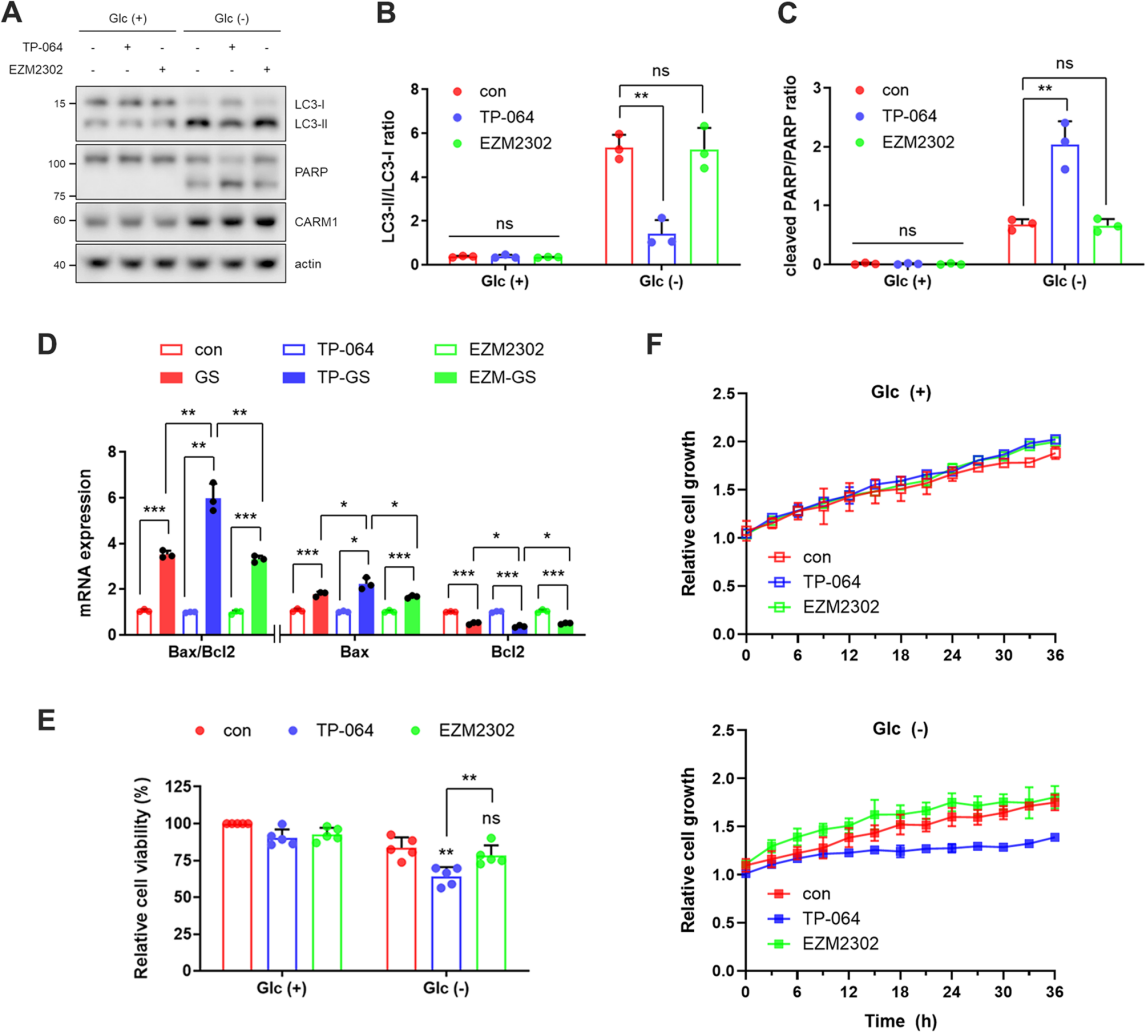
TP-064, but not EZM2302, sensitizes to energy stress by inhibiting autophagy. (**A**) Representative immunoblot of LC3 and PARP from three independent experiments. (**B**) Quantification of the LC3-II/LC3-I ratio and (**C**) cleaved PARP levels from panel (**A**) (*n* = 3). (**D**) mRNA expression levels of apoptosis-related genes (*n* = 3). (**E**) Relative cell viability measured by MTT assay (*n* = 5) in MEF cells treated with CARM1 inhibitors under 12 h glucose deprivation. ‘n’ indicates the number of independent experiments performed. (**F**) Relative cell growth was quantified from long-term live cell imaging of MEF cells pretreated with TP-064 or EZM2302 and cultured in media with or without glucose (*n* = 3)

Autophagy is a well-established pro-survival mechanism that suppresses apoptosis, and its sustained inhibition or functional collapse under stress can lead to apoptosis as a fail-safe response (Levine et al. 2005; Rubinstein et al. 2012). We therefore examined whether the two CARM1 inhibitors differentially modulate apoptosis under energy stress conditions. As expected, our results show that TP-064, but not EZM2302, markedly enhanced apoptosis, as indicated by increased PARP cleavage (Fig. 3A), increased Bax/Bcl-2 ratio (Fig. 3B), and reduced cell viability (Fig. 3C). In addition, we further confirmed these findings using a long-term live cell imaging system. Under glucose-deprived metabolic stress conditions, only TP-064, but not EZM2302, markedly suppressed cell growth (Fig. 3F and S8). Collectively, these findings suggest that TP-064-mediated inhibition of autophagy impairs the cellular ability to tolerate energy stress and shifts the balance toward apoptotic cell death.

**Fig. 4:**
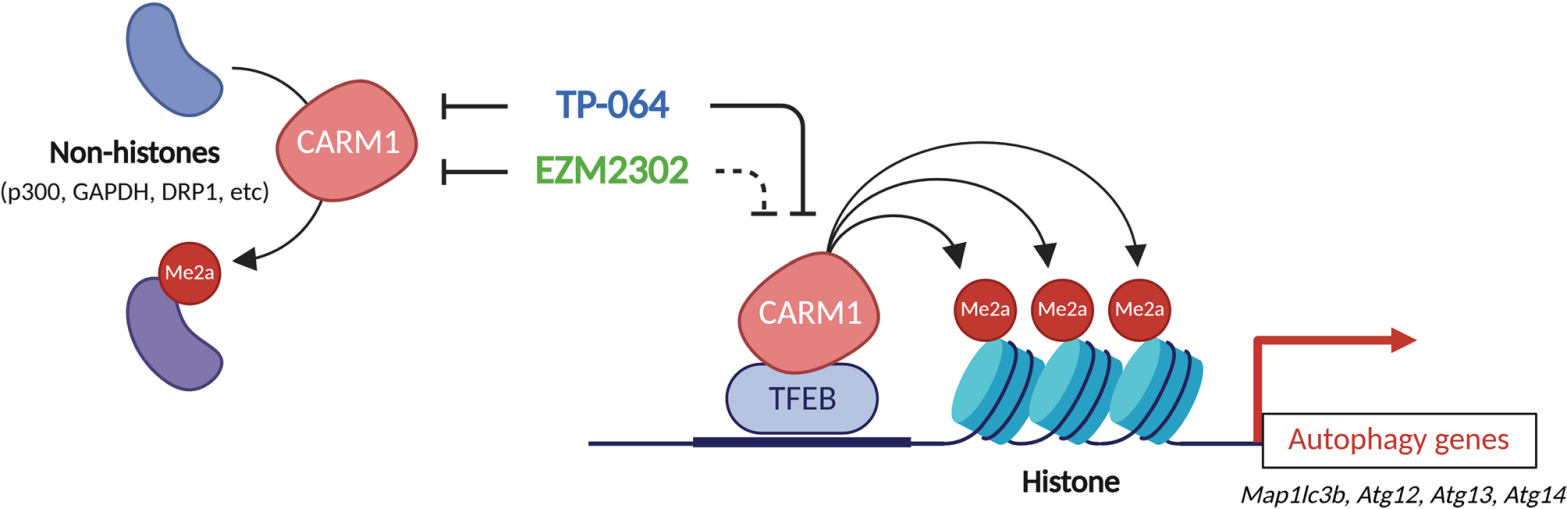
Functional differences between TP-064 and EZM2302. Our findings show a substrate-selective mechanism of action: TP-064 targets both nuclear and cytoplasmic functions, whereas EZM2302 selectively inhibits non-histone methylation events

Our findings reveal that TP-064 and EZM2302, despite being potent CARM1 inhibitors with comparable IC₅₀ values, exhibit distinct substrate preferences and cellular effects (Fig. 4). This divergence is likely due to differences in their physicochemical properties, alteration of substrate-binding affinity, or subcellular distribution. TP-064 effectively inhibits histone methylation (e.g., H3R26me2a, H3R17me2a), leading to suppression of CARM1-mediated transcriptional programs—including autophagy gene expression—thereby impairing autophagic flux and sensitizing cells to energy stress. In contrast, EZM2302 spares histone methylation and primarily targets non-histone substrates, preserving autophagic function and cellular stress resistance. Prior studies have demonstrated mechanistic differences in how these inhibitors engage CARM1: TP-064 cooperatively binds with SAM (Nakayama et al. 2018), whereas EZM2302 stabilizes an inactive CARM1–SAH complex (Drew et al. 2017). These distinct binding modes likely underlie their differential substrate specificities, particularly with respect to histone versus non-histone targets. Such mechanistic divergence has important therapeutic implications. For example, TP-064 may be more effective in transcription-driven cancers (e.g., breast cancer, leukemias), where repression of oncogenic transcription is desirable. EZM2302, by selectively targeting non-histone substrates of CARM1, could be better suited for diseases involving cytoplasmic signaling imbalances. Together, our data underscore the need for mechanism-based selection of CARM1 inhibitors, rather than relying solely on enzymatic potency. Furthermore, the distinct cellular effects of TP-064 and EZM2302 support the development of substrate- or compartment-specific inhibitors that maximize therapeutic benefit while minimizing off-target consequences. Their divergent impact on autophagy and apoptosis pathways also opens new avenues for rational combination therapies in cancer and metabolic disease.

In summary, this study highlights the necessity of a mechanistically informed, context-specific approach to deploying CARM1 inhibitors in both basic research and translational settings. By dissecting the substrate-specific and functional profiles of TP-064 and EZM2302, we provide a conceptual and practical framework for tailoring CARM1-targeted interventions to distinct biological and therapeutic scenarios.

## Supplementary Information

Supplementary Material 1.

## Data Availability

No datasets were generated or analysed during the current study.

## Abbreviations

ADMA: Asymmetric dimethylarginine
AMPK: AMP-activated protein kinase
CARM1: Coactivator-associated arginine methyltransferase 1
DRP1: Dynamin-related protein 1
GAPDH: Glyceraldehyde-3-phosphate dehydrogenase
MEF: Mouse embryonic fibroblast
MMA: Monomethylarginine
PARP: Poly (ADP-ribose) polymerase
PRMT: Protein arginine methyltransferase
SAH: S-adenosylhomocysteine
SAM: S-adenosylmethionine
SDMA: Symmetric dimethylarginine
