# Supplementary material for: Context-specific applications of CARM1 inhibitors: functional profiles of EZM2302 and TP-064
Source: Mol Med. 2025 Oct 31;31:322. doi: 10.1186/s10020-025-01388-y (PMC12576987; doi:10.1186/s10020-025-01388-y)
Supplement: Supplementary file 1 — Supplementary Material 1. [file 10020_2025_1388_MOESM1_ESM.docx]

**Supplementary Information**

**Context-Specific Applications of CARM1 Inhibitors:
Functional Profiles of EZM2302 and TP-064**

**­­**

Yena Cho^1,2^ and Yong Kee Kim^1,2,^*

^1^Muscle Physiome Research Center and Research Institute of Pharmaceutical Sciences, Sookmyung Women’s University, Seoul 04310, Republic of Korea

^2^College of Pharmacy, Sookmyung Women’s University, Seoul 04310, Republic of Korea

*Correspondence to: Yong Kee Kim, Ph.D., E-mail: yksnbk@sookmyung.ac.kr, Tel: +82-2-2077-7688, Fax.: +82-2-710-9871

**
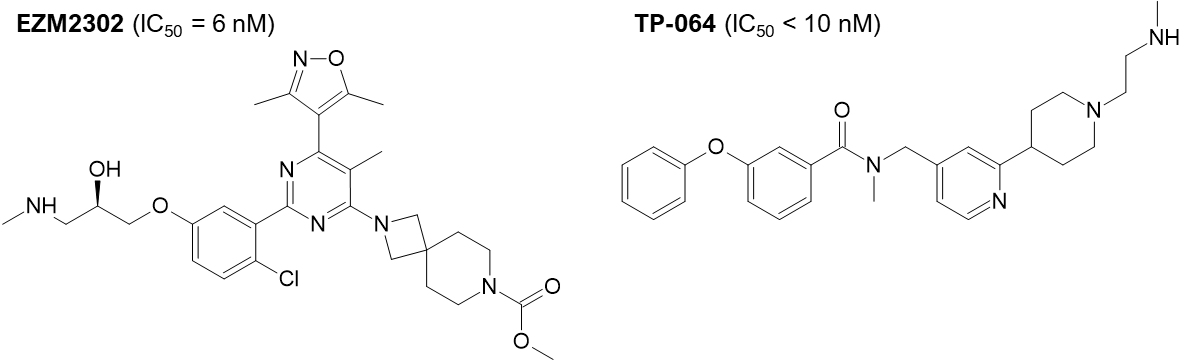
**

**Fig. S1. Chemical structures of CARM1 inhibitors.**

**
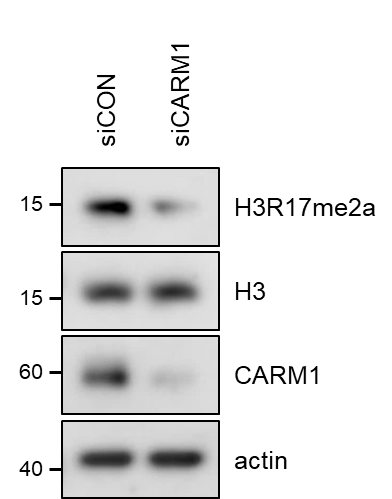
**

**Fig. S2. Western blot analysis of H3R17me2a levels after CARM1 knockdown.** After incubation with CARM1 siRNA for 72 h, cell lysates were prepared and analyzed for H3R17me2a levels by western blotting.

**
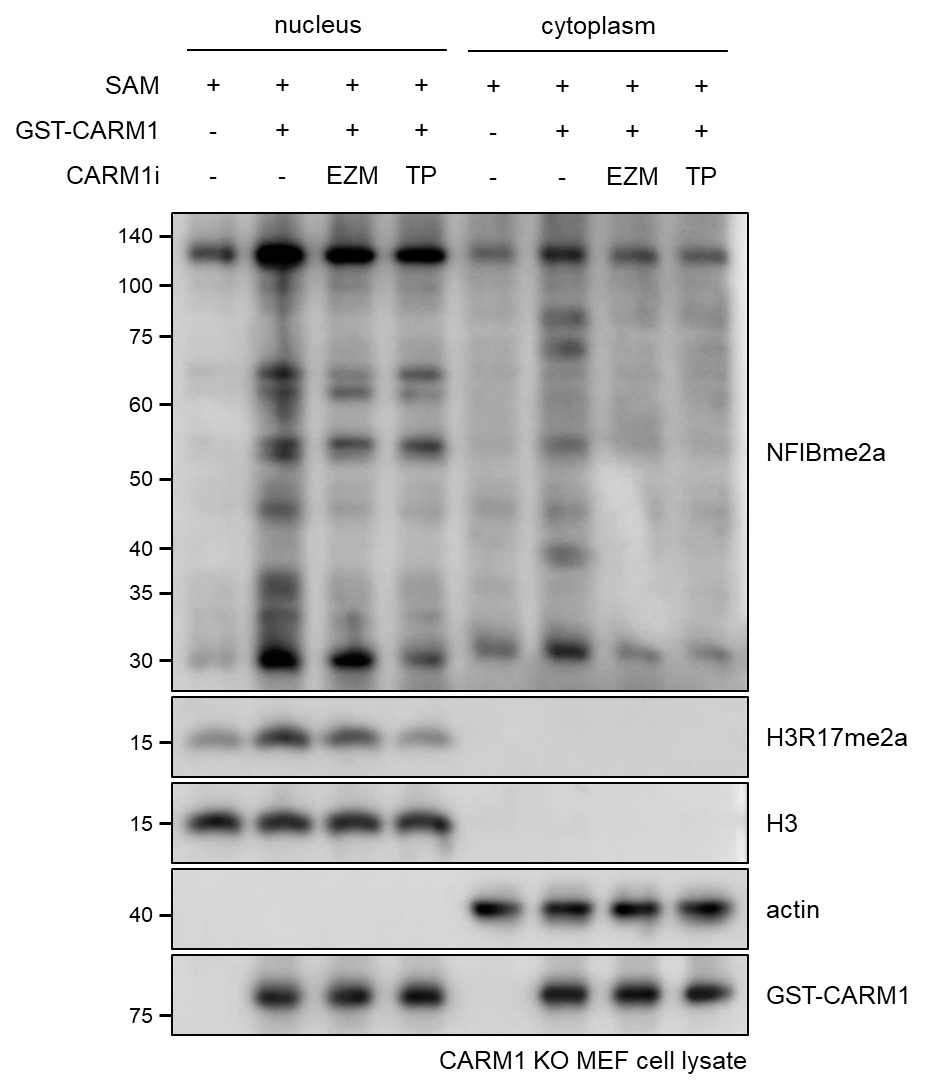
**

**Fig. S3. *In vitro* methylation assay of nuclear and cytoplasmic fractions from CARM1 KO MEFs.** CARM KO MEF cell lysates were fractionated into nuclear and cytoplasmic components. Each fraction was incubated *in vitro* with 1 μM SAM and GST-CARM1 protein in the presence or absence of CARM1 inhibitors (10 nM) for 1 h at 37 °C. Methylation levels in the nuclear and cytoplasmic fractions were assessed by western blot using NFIBme2a and H3R17me2a antibodies.

**
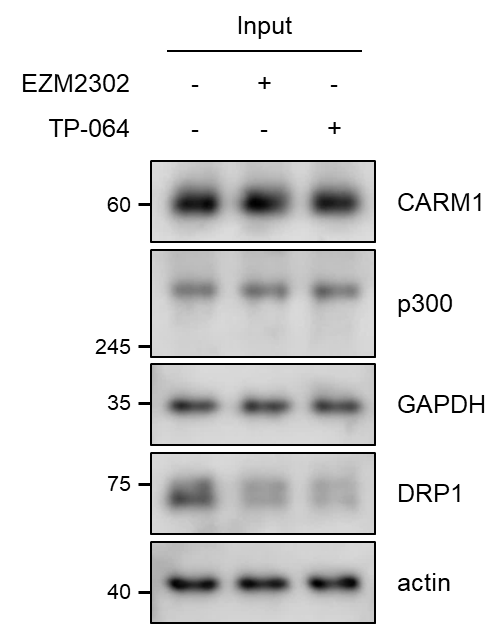
**

**Fig. S4. Western blot analysis of p300, GAPDH, and DRP1 levels after CARM1 inhibitor treatment.** After treatment with CARM1 inhibitors (1 μM, 72 h), cell lysates were prepared and subjected to immunoprecipitation using anti-p300, anti-GAPDH, or anti-DRP1 antibodies. The expression levels of p300, GAPDH, and DRP1 were examined by western blotting as input controls for Fig. 1E.


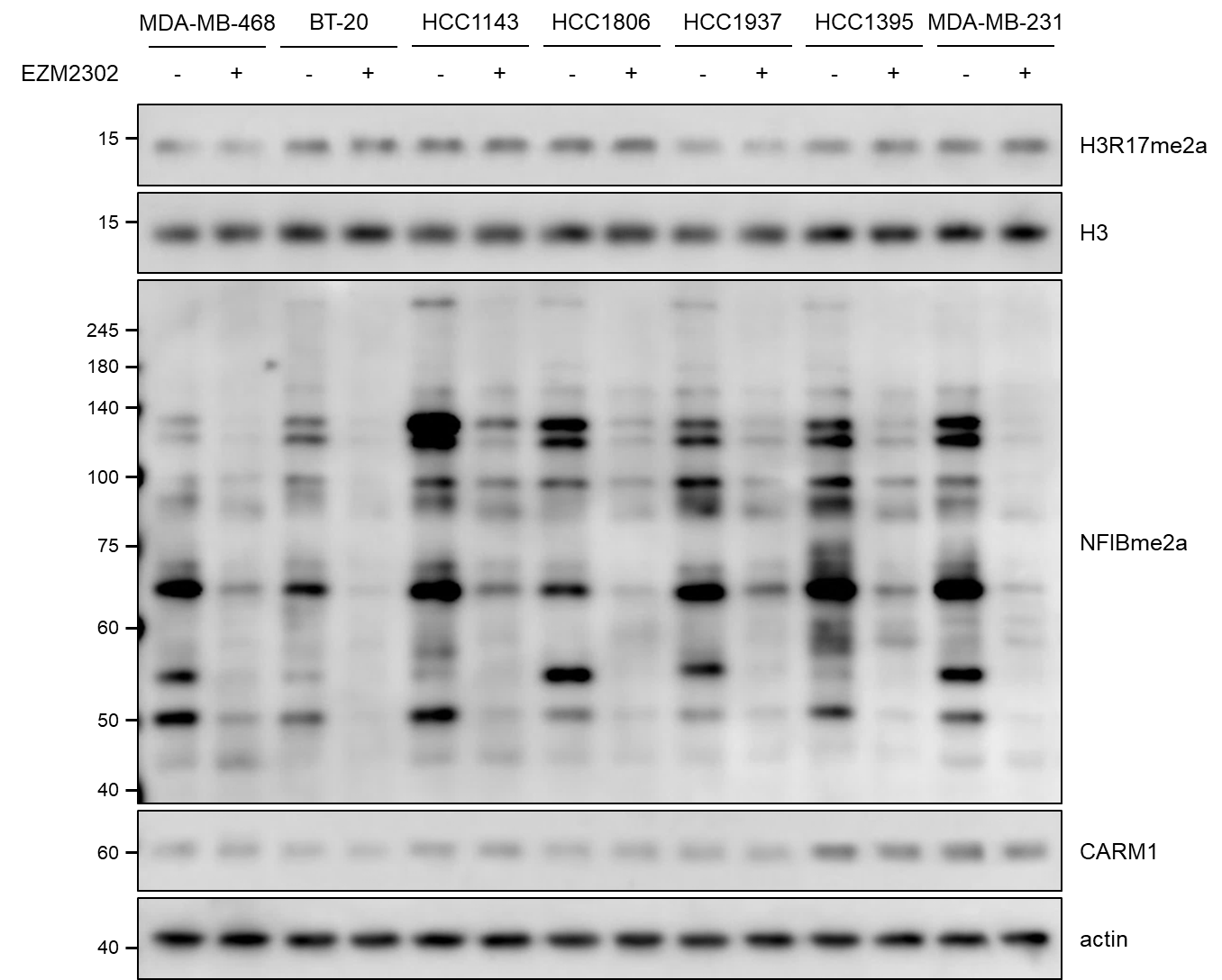


**Fig. S5. Western blot analysis of histone and CARM1 substrate methylation after EZM2302 treatment in various breast cancer cell lines.** After treatment with EZM2302 (1 μM, 72 h), cell lysates were prepared and analyzed for H3R17me2a and pan-CARM1 substrate methylation levels by western blotting.


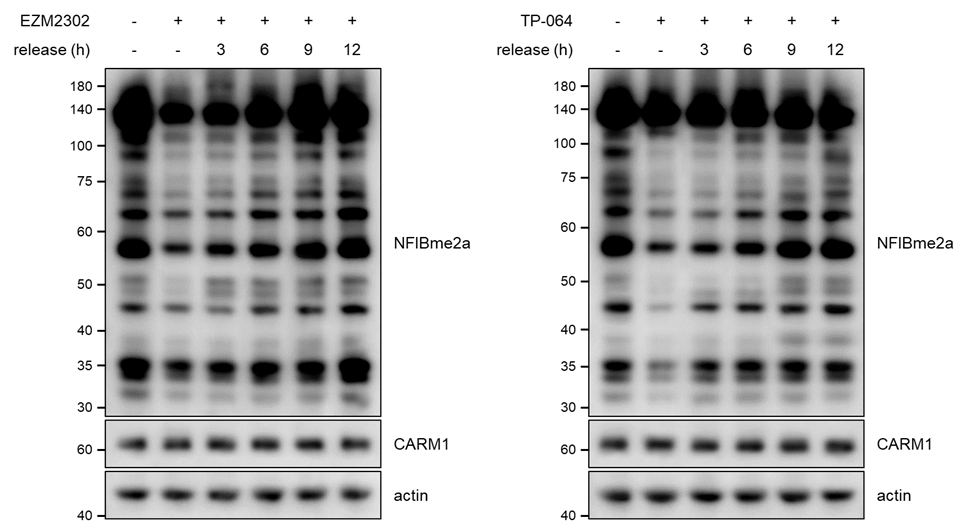


**Fig. S6. Time-course analysis of reversible recovery of CARM1 activity after treatment with EZM2302 and TP-064.** MEF cells were treated with EZM2302 or TP-064 for 24 h, followed by incubation in fresh medium for the indicated time periods. CARM1 substrate levels were then analyzed by western blotting.

**
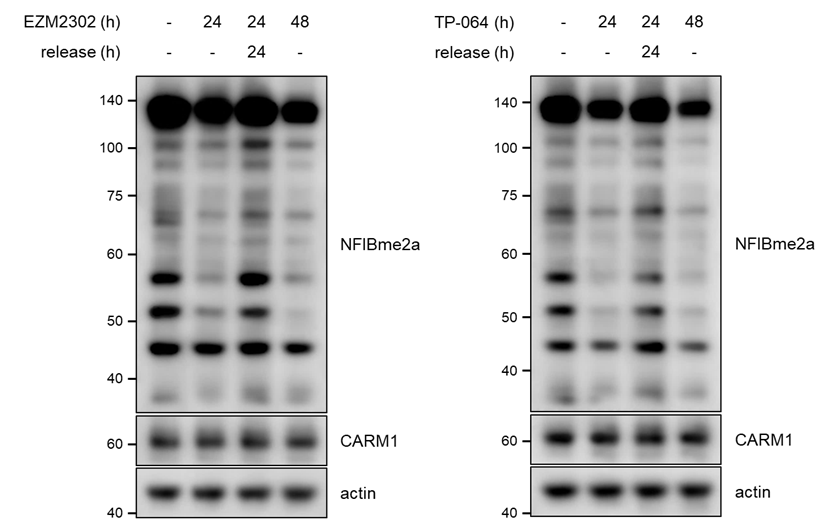
**

**Fig. S7. Reversible recovery of CARM1 activity after treatment with EZM2302 and TP-064.** HEK293T cells were treated with EZM2302 or TP-064 for 24 h, followed by an additional 24 h incubation in fresh medium. CARM1 substrate levels were then analyzed by western blotting.


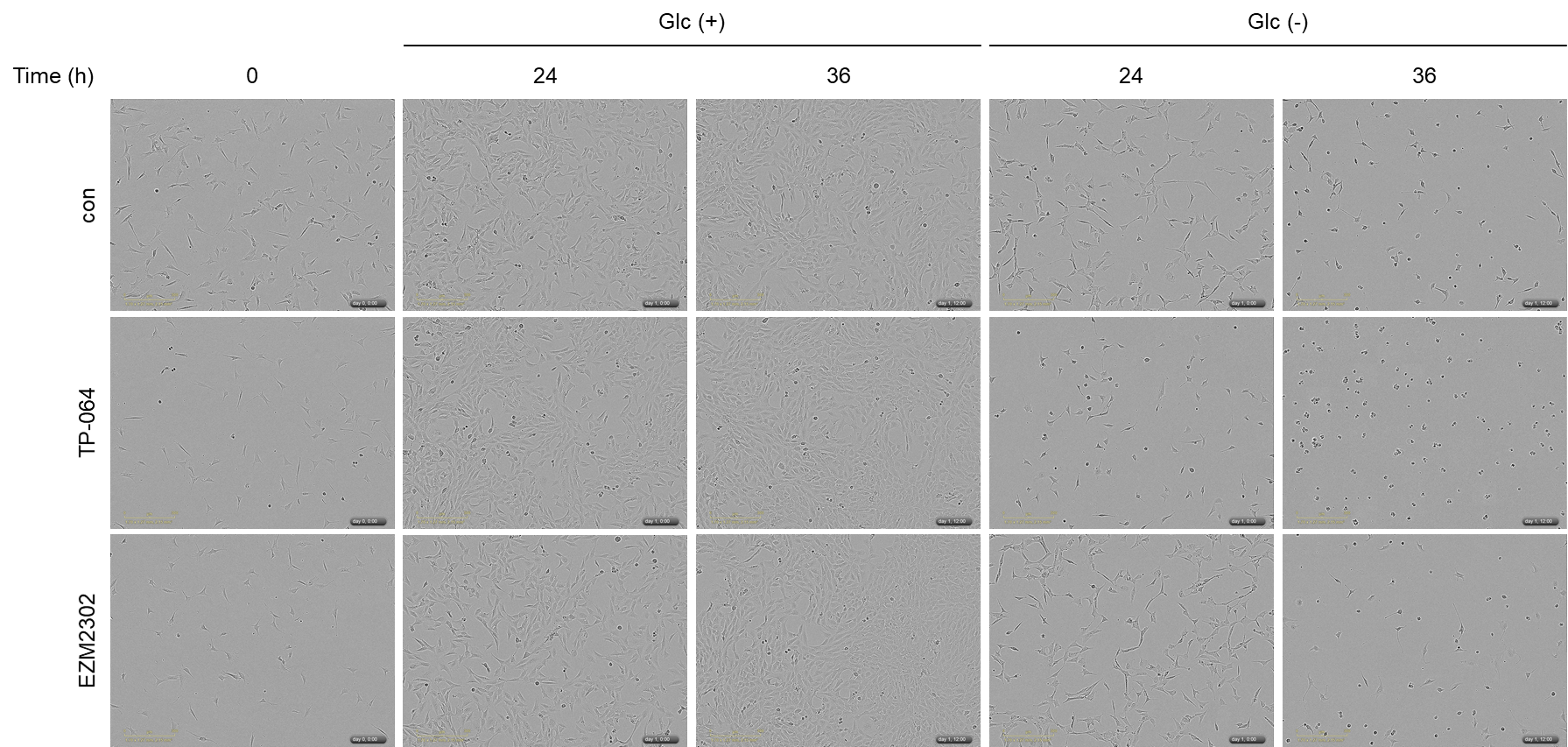


**Fig. S8. Representative live cell images.** MEF cells pretreated with TP-064 or EZM2302 were incubated in media with or without glucose for the indicated time periods. Relative cell growth was quantified and shown in Fig. 3F.
